# DET induces apoptosis and suppresses tumor invasion in glioma cells via PI3K/AKT pathway

**DOI:** 10.3389/fonc.2024.1528454

**Published:** 2025-01-09

**Authors:** Rui Zhao, Mengran Wang, Zeyu Wu, Panpan Zhao, Huiling Dong, Yue Su, Chenghui Zhao, Min Qi, Shizhang Ling, Xiaochun Jiang

**Affiliations:** ^1^ The Translational Research Institute for Neurological Disorders of Wannan Medical College, Department of Neurosurgery, The First Affiliated Hospital of Wannan Medical College (Yijishan Hospital of Wannan Medical College), Wuhu, Anhui, China; ^2^ The Institutes of Brain Science, Wannan Medical College, Higher Education Park, Wuhu, Anhui, China

**Keywords:** glioma, cell proliferation, cell apoptosis, Pl3K, cell invasion

## Abstract

**Introduction:**

Gliomas, particularly glioblastomas (GBM), are highly aggressive with a poor prognosis and low survival rate. Currently, deoxyelephantopin (DET) has shown promising anti-inflammatory and anti-tumor effects. Using clinical prognostic analysis, molecular docking, and network pharmacology, this study aims to explore the primary targets and signaling pathways to identify novel GBM treatment approaches.

**Methods:**

Using PharmMapper, the chemical structure of DET was examined for possible targets after being acquired from PubChem. GBM-related targets were obtained through multi-omics approaches. A protein-protein interaction (PPI) network was constructed using Cytoscape and STRING, and target binding was evaluated through molecular docking. Enrichment analysis was conducted using Metascape. The effects of DET on GBM cell invasion, apoptosis, and proliferation were assessed through *in vitro* assays, including Transwell, EDU, CCK8, and flow cytometry. Western blot analysis was performed to examine the components of the PI3K/AKT signaling pathway.

**Results:**

Among the sixty-four shared targets identified, JUN and CCND1 were the most frequently observed. Enrichment analysis demonstrated that DET influenced the MAPK and PI3K/AKT signaling pathways. In Transwell assays, DET significantly inhibited the invasive behavior of glioma cells. Western blot analysis further confirmed the downregulation of EGFR, JUN, and PI3K/AKT.

**Conclusion:**

DET inhibits GBM cell invasion, proliferation, and apoptosis via modulating the PI3K/AKT signaling pathway, highlighting its potential as a novel therapeutic strategy for GBM treatment.

## 1 Introduction

Gliomas account for approximately 80% of malignant brain tumors and 30% of all primary brain tumors, arising from genetic mutations in neural stem or progenitor cells (1). The World Health Organization’s (WHO) grading system divides gliomas into four histological categories. Pilocytic astrocytoma, the least malignant form of astrocytoma, typically has a life expectancy of 5 to 10 years (WHO grade I). GBM is classified as a World Health Organization (WHO) grade IV tumor and is the most common and aggressive form of malignant glioma, accounting for 57.3% of cases. Its highly invasive characteristics, rapid progression, and frequent recurrence contribute to a 5-year survival rate of only 6.8%. The median survival duration following diagnosis is approximately 15 months (2).

Multiple factors influence the progression and therapeutic outcomes of tumors, including changes in the immune microenvironment (3–6), drug resistance (7), and alterations in key signaling pathways, such as PI3K/AKT pathway (8, 9). In order to increase patient survival and improve prognosis, current therapeutic options for gliomas include radiation, temozolomide chemotherapy, surgery, and tailored treatment techniques (10). However, glioblastomas, a subtype of gliomas, is highly susceptible to recurrence and exhibit resistance to subsequent therapies, which accelerates disease progression and often results in limited treatment efficacy (11). In order to improve patient outcomes and survival, additional research is desperately needed that focuses on creating novel treatment plans or finding anti-cancer medications that work better.

The issue of drug resistance in gliomas is currently being tackled through multi-targeted therapies, including those aimed at the epidermal growth factor receptor (EGFR) and its downstream signaling pathways, such as PI3K (12). The wide range of anti-inflammatory and anti-cancer properties of DET have attracted interest (13). DET has been shown to inhibit various malignancies, such as liver, breast, and cervical cancers, through multiple molecular pathways, including the NF-κB signaling cascade (14–18), and has also demonstrated efficacy in treating non-cancerous conditions like fulminant hepatitis (19). However, the precise effects and underlying regulatory mechanisms of DET in gliomas remain poorly understood, hindering its clinical application and underscoring the necessity for further research.

Using network pharmacology, molecular docking, and clinical prognostic analysis, this study sought to determine the major molecular targets and signaling pathways implicated in the therapeutic actions of DET against glioma. U87-MG and T98G glioblastoma (GBM) cell lines were used in *in vitro* tests to evaluate the effects of DET on cell invasion, proliferation, and the expression of key targets and signaling pathway elements. To assess DET’s *in vivo* anti-glioma activities, a xenograft tumor model in naked mice was also developed. As far as we are aware, this is the first study to show that DET has the ability to treat gliomas both *in vitro* and *in vivo*. This lays the groundwork for further studies into the clinical use of DET in glioma treatment.

## 2 Materials and methods

### 2.1 Reagents and materials

Deoxyelephantopin (DET) (HY-N2491, purity ≥99%) was purchased from MCE (Shanghai, China). DET was dissolved in DMSO (Sigma-Aldrich, St. Louis, MO, United States) for storage. The Cell Counting Kit-8 (KGA9305-1000), kFluor488-EDU Assay Cell Proliferation Detection Kit (KGA9602-100), and Annexin V-FITC Apoptosis Detection Kit (KGA1102-100) were purchased from KGI Biotechnology Development Co. (Nanjing, China). The antibodies against PI3K (cst4257), AKT (cst4691), and phosphorylated-AKT (cst4060) were purchased from Cell Signaling Technology, Inc. (Danvers, MA, USA). Goat anti-rabbit IgG H&L (HRP polymer) (ab214880), KI67 (ab16667), EGFR (ab52894), and C-JUN (ab40766) antibodies were purchased from Abcam (Cambridge, MA, United Kingdom). β-tubulin (AF4351), GAPDH (AF7021), and goat anti-rabbit IgG (H+L) HRP antibodies (S0001) were purchased from Affinity Biosciences (Liyang, China). Transwell inserts (3428) with a pore size of 8 microns and Matrigel (354234) were obtained from Corning (Corning, NY, USA).

### 2.2 Gene ontology and KEGG enrichment

Potential signaling pathways were further explored using KEGG enrichment analysis. Additional analysis and visualization of the GO and KEGG data were conducted to gain deeper insights. Prognosis analysis of core targets was performed using the survival package in R (version 4.2.2) based on the collected data. Kaplan-Meier survival curves were generated to investigate the relationship between the expression levels of key targets and the survival outcomes of glioma patients (20–22).

### 2.3 Molecular docking analysis

The Protein Data Bank (PDB) (https://www.rcsb.org/) provided the molecular structures of the EGFR (PDBID: 5UGB) and JUN (PDBID: 2P33) proteins. Using Pymol software, the proteins and DET were then produced by performing hydrogenation and dehydrogenation methods, which involved removing ligands and rotatable torsion bonds. After this preparation, molecular docking simulations were conducted using AutoDock (version 1.5.7), and the binding affinities were evaluated to assess the strength of the interactions between DET and the target proteins.

### 2.4 Cell viability assessment

We purchased U87-MG and T98G from the Cell Bank in Shanghai, China. T98G and U87-MG cells were seeded at a density of 5,000 cells per well onto 96-well plates for the tests. The cells were subjected to different DET concentrations (0, 10, 20, or 40 µM) after a 24-hour period. The CCK-8 reagent was added to each well 24 and 48 hours after treatment. After the reagent had been incubated for two hours in a cell culture incubator, the optical density was then measured at 450 nm using a BioTek microplate reader (Vermont, USA).

### 2.5 Cell proliferation assay

The kFluor488-EDU assay was used to measure cell proliferation. After being seeded onto 96-well plates, U87-MG and T98G cells were grown until they reached the exponential phase. After that, the cells received a 24-hour DET treatment. After that, proliferating cells were labeled by adding EDU to the culture media for two hours. After fixing the cells for half an hour, the extra solution was drained off. After adding the Click Reaction Solution, it was incubated for 20 minutes at 25°C. After three PBS washes, the cells were incubated for fifteen minutes with 5 μg/mL Hoechst33342.

### 2.6 Annexin V-FITC/PI staining

The T98G and U87-MG glioblastoma cell lines were seeded on 6-well plates and exposed to varying concentrations of DET for 24 hours. Apoptotic cell death was quantified using the Annexin V-FITC/PI Apoptosis Detection Kit. Following treatment, cells were detached using EDTA-free trypsin, and residual trypsin was removed by washing with PBS. Single-cell suspensions were prepared by resuspending the cells in 500 μL of Binding Buffer (23). The suspensions were incubated for 15 minutes at room temperature in the dark with Annexin V-FITC and PI. Apoptosis was assessed via flow cytometry.

### 2.7 Cell invasion

Matrigel (354234, Corning) and Transwell inserts with an 8-micron size were used to assess the effect of DET on glioma cell invasion. The glioma cell lines were planted in the upper compartment with 300 μL of serum-free media after being exposed to different DET doses. 650 μL of medium supplemented with 10% serum was introduced to the lower chamber. 100 μL of Matrigel was applied beforehand to each Transwell insert. The media was disposed of after the cells had been incubated for twenty-four hours. After being fixed for 30 minutes with 4% paraformaldehyde, the cells on the bottom surface of the Transwell membrane were stained with 0.5% crystal violet (24). The number of invading cells was quantified by taking pictures of the labeled cells using fluorescent microscopy.

### 2.8 Western blot

DET was administered to T98G and U87-MG cells for a whole day. RIPA buffer with protease and phosphatase inhibitors was used to extract the proteins, and the BCA assay was used to measure the protein concentration. After being denatured for eight minutes at 98°C using 5× SDS-PAGE buffer, the lysates were separated using 10% SDS-PAGE and then transferred onto PVDF membranes with a pore size of 0.45 µm. Following two hours of blocking with 5% BSA, membranes were incubated with a primary antibody at 4°C for the whole night, followed by an hour incubation at room temperature with a secondary antibody (25). An improved ECL detection kit was used to observe the protein bands, and ImageJ software was used for analysis.

### 2.9 Creation and assessment of GBM xenograft model

This study was approved by the Ethics Committee at Yijishan Hospital. Four-week-old male nude mice, sourced from the Experimental Animal Center of Hangzhou Medical College, were housed in specific-pathogen-free (SPF) conditions. A glioblastoma xenograft model was established by subcutaneously injecting U87-MG glioma cells (2×10^7^ cells per mouse) into the left lower abdomen of each mouse. The mice were then randomly divided into two groups of five. One week after inoculation, the experimental group received intraperitoneal injections of DET (10 mg/kg of body weight, administered every other day), while the control group was given saline injections. Tumor growth was monitored by measuring the longest and shortest tumor diameters every three days for 18 days. On day 18, the mice were humanely euthanized, and tumors, livers, and kidneys were collected for further analysis.

### 2.10 Statistical analyses

The experimental results were expressed as the mean ± standard deviation. Plotting and statistical analyses were performed using GraphPad Prism 8.0 (GraphPad Software, San Diego, CA, USA) (26). Group comparisons were conducted using one-way analysis of variance (ANOVA) or the Student’s t-test, with *P* < 0.05 considered statistically significant.

## 3 Results

### 3.1 Venn analysis and database mining to find possible DET and glioma cross-targets

**Figure 1:**
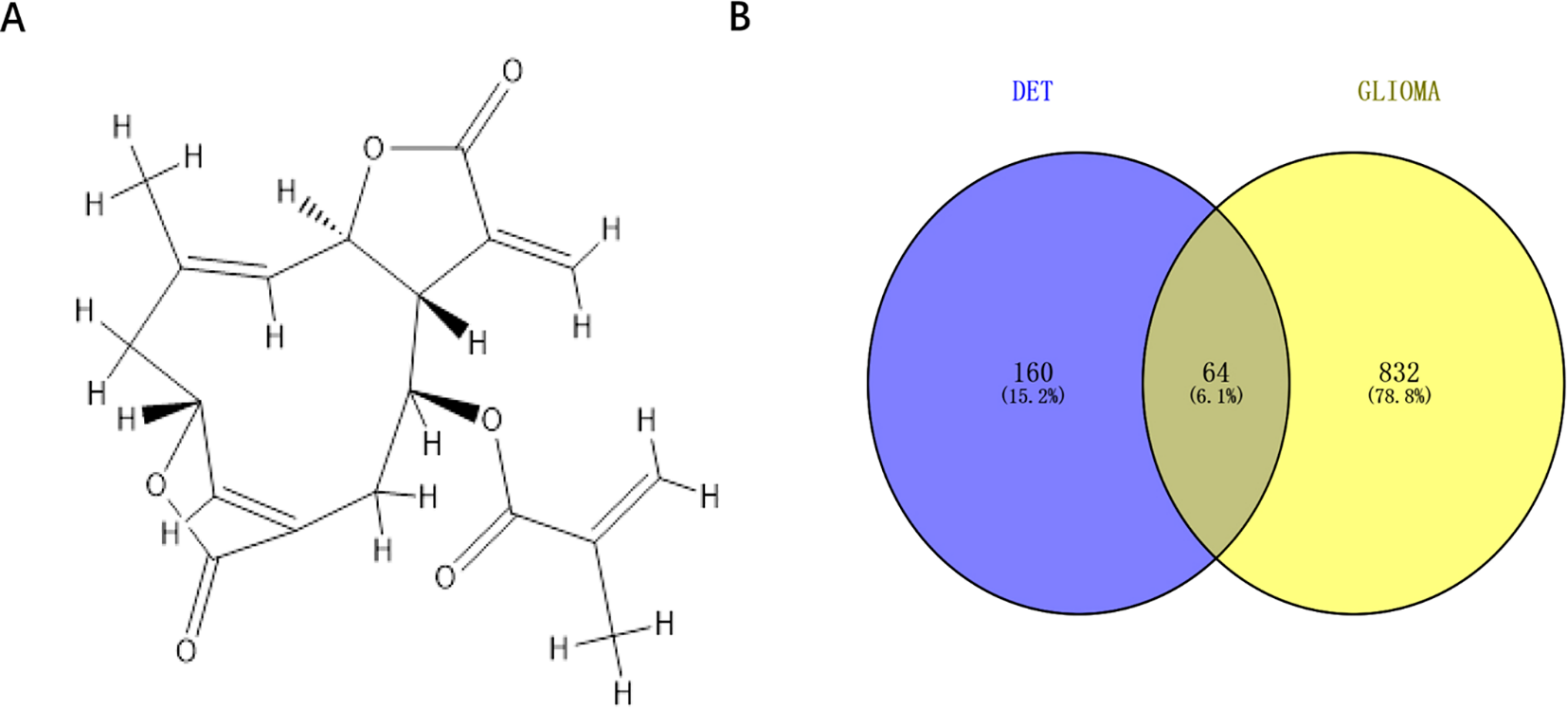
Cross-target acquisition. **(A)** The chemical structure of DET in two dimensions. **(B)** Venn diagram of genes linked to gliomas and DET targets.

The chemical structure of DET was obtained from the PubChem Compound database (CID: 6325056) (**Figure 1A**). There were totally 224 possible DET targets found from the PharmMapper (default settings), TCMSP (default settings), and SwissTargetPrediction (probability > 0) databases. Additionally, 767, 60, 17 and 34 glioma targets were identified from the GeneCard (relevance score ≥ 2), TherapeuticTarget (default settings), DisGeNET (score > 0), and OMIM (default settings) databases, respectively. The combined results of these four databases yielded 896 glioma targets after false-positive targets and duplicate values were eliminated. As seen in **Figure 1B**, 64 common targets were obtained when the DET and glioma cross-targets were uploaded into Venny2.1.

### 3.2 Identification of EGFR and JUN as core therapeutic targets

**Figure 2:**
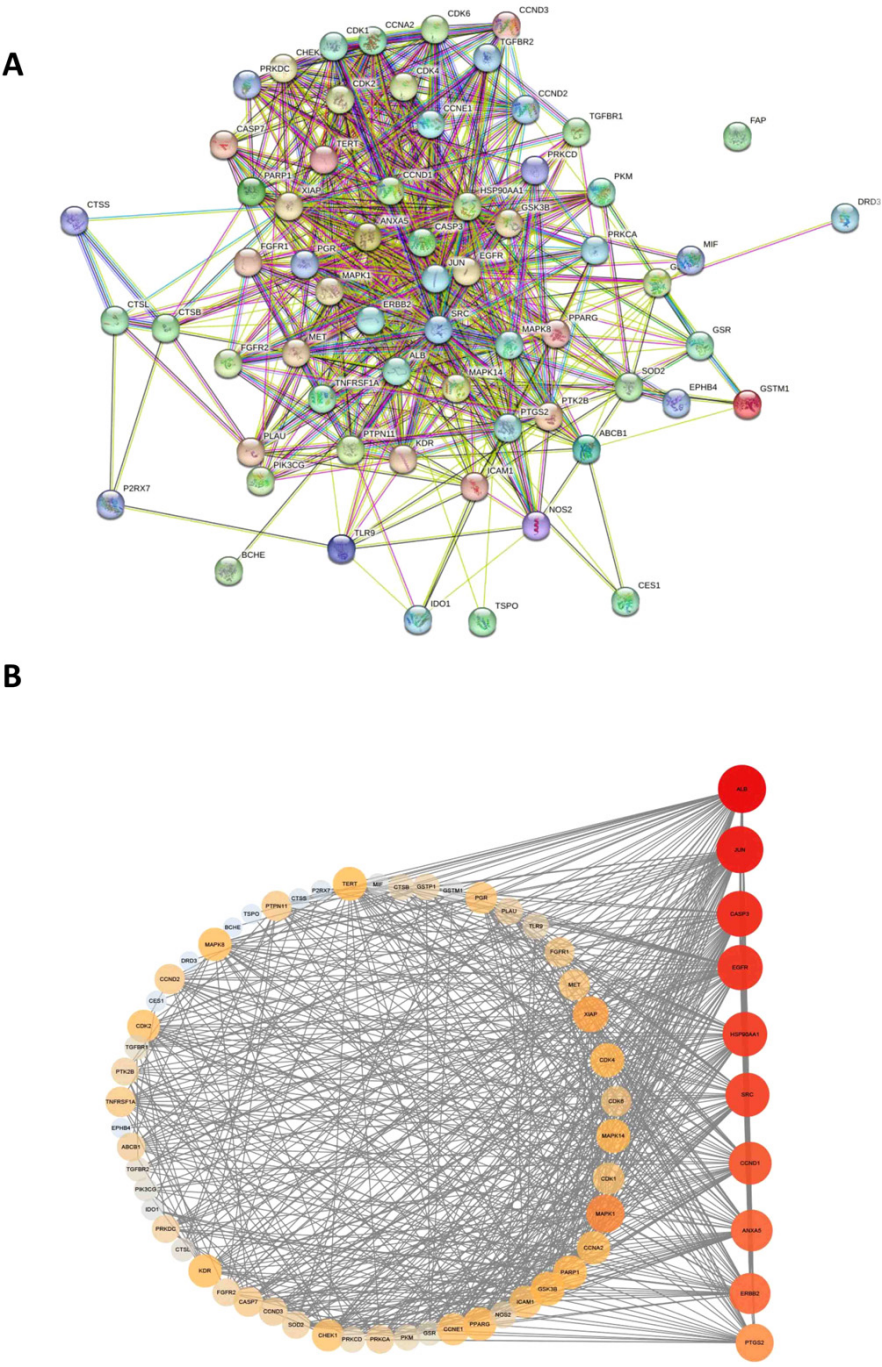
PPI network construction. **(A)** By uploading intersecting genes from the STRING database, a protein-protein interaction (PPI) network was created. **(B)** Cytoscape was used to rank the top 10 hub genes of the PPI network (the vertical list of genes displayed on the right side of the panel). The higher the association between a gene and glioma, the more reddish the gene’s circle appears in the vertical list of genes. The higher the association between a gene and the other genes displayed in the circle on the left side of the panel, the bigger the circle of that gene is in the vertical list of genes.

After importing the acquired cross-targets into the STRING database (confidence > 0.5), a PPI network of 64 nodes and 726 edges were built (**Figure 2A**). After that, Cytoscape was used to import the acquired data files for analysis and visualization. EGFR and JUN were identified as the two main therapeutic targets (**Figure 2B**). This implies that these two possible targets are important components of DET’s mode of action when treating gliomas.

### 3.3 Identification of molecular mechanism

**Figure 3:**
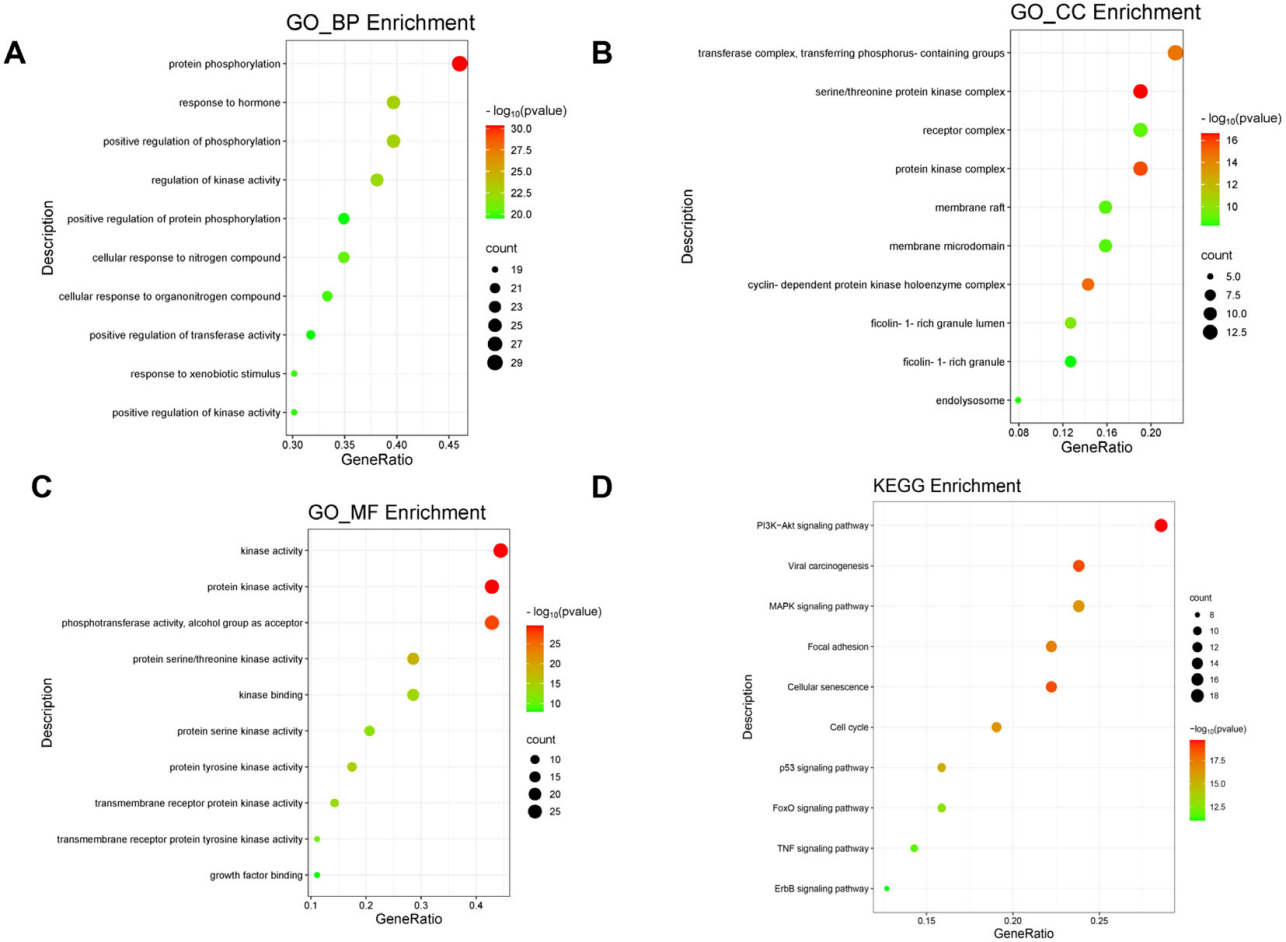
Utilizing the GO and KEGG pathways, overlapping genes are examined. **(A-C)** Bubble plot showing the GO enrichment analysis’s top ten biological processes (BP), cellular components (CC), and molecular functions (MF), respectively. **(D)** Bubble plot of the KEGG pathway enrichment’s top ten signaling pathways.

After performing GO and KEGG pathway analyses on the 64 cross-target genes of DET in GBM using the Metascape platform, the top 10 keywords were selected for the creation of a bubble plot (**Figures 3A-C**). The biological process (BP) enrichment highlighted DET’s involvement in hormone response, positive regulation of phosphorylation, modulation of kinase activity, and protein phosphorylation (**Figure 3A**). Cellular component (CC) enrichment identified key complexes such as the serine/threonine protein kinase complex, phosphotransferase complex, and receptor complex, suggesting that DET’s anti-tumor effects in GBM are linked to its influence on phosphotransferase functions, protein kinase activity, and kinase regulation (**Figure 3B**). In **Figure 3C**, we further elaborate on the molecular roles involved. KEGG pathway analysis revealed 150 pathways, with the PI3K/AKT signaling pathway emerging as a crucial mechanism through which DET exerts its therapeutic effects in GBM (**Figure 3D**).

### 3.4 Molecular docking of DET

**Figure 4:**
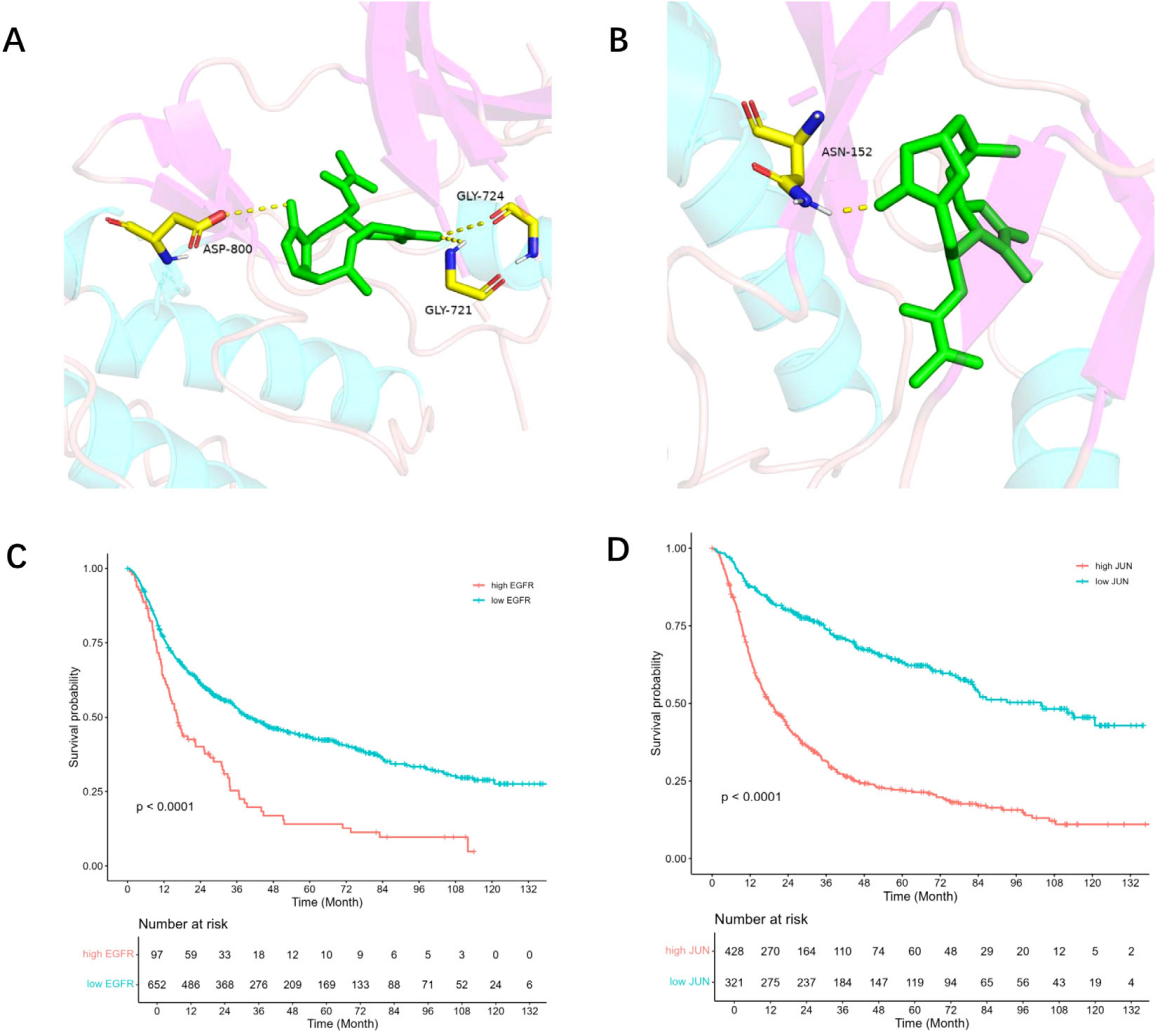
Core target survival analysis and molecular docking. **(A)** DET and EGFR protein molecules are docked together; EGFR and DET are shown by green and brown hues, respectively. **(B)** DET docking findings with JUN protein molecule; JUN and DET are shown by green and brown hues, respectively. **(C)** The association between glioma patients’ prognosis survival and the EGFR gene’s expression level. Patients with high EGFR expression are shown by the red line, whereas those with low expression are shown by the green line. **(D)** The association between glioma patients’ prognosis survival and JUN gene expression. Patients with high JUN expression are shown by the red line, whereas those with low JUN expression are represented by the green line.

Molecular docking studies of EGFR and JUN, the two primary targets of DET, were conducted using AutoDock software (**Figures 4A, B**). Affinity scores, which are used to evaluate the stability of ligand-receptor interactions, indicated that values below -5.0 kcal/mol typically represent strong binding. The results demonstrated that DET exhibits a significant affinity for both EGFR and JUN.

### 3.5 EGFR and JUN expression in GBM prognosis

The CGGA database was used for prognostic analysis in order to evaluate the impact of core targets on the prognosis of GBM. Survival analysis showed that patients with higher expression levels of EGFR and JUN had a statistically significant difference (*P* < 0.001) in overall survival when compared to those with lower expression levels (**Figures 4C, D**). These results suggest that EGFR and JUN, two important DET targets, play a crucial role in the development of gliomas and have a major influence on the prognosis of glioma patients.

### 3.6 DET inhibits glioma cell proliferation

**Figure 5:**
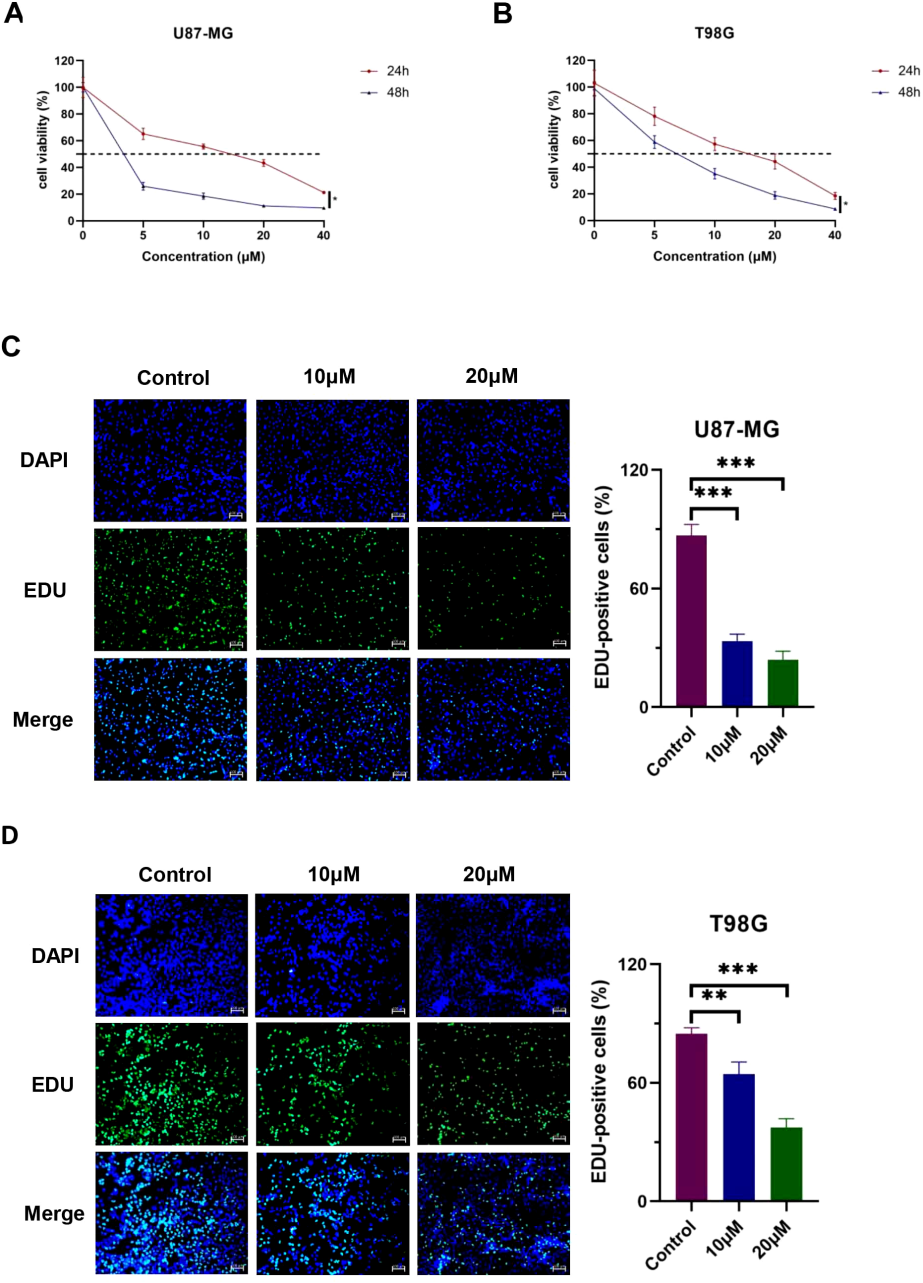
DET stops T98G and U87-MG cells from proliferating. **(A, B)** DET significantly and dose-dependently inhibited the viability of both cell lines, according to the CCK-8 test. **(C, D)** DET significantly inhibited the proliferation of T98G and U87-MG cells, according to the EDU test. *P<0.05, **P<0.01, ***P<0.001.

We used the CCK-8 test to measure the proliferative capabilities of GBM cells after DET exposure in order to ascertain the effect of DET on the proliferation of glioma cell lines. As seen in **Figures 5A, B**, this study revealed a significant decline in the proliferation rates of T98G and U87-MG cells, which correlated adversely with exposure duration and dose. To further measure DET’s anti-proliferative effects, we also used EDU-DNA tests. Compared to the control group, a significant decrease in the percentage of EDU-positive cells was noted as the DET concentration rose (**Figures 5C, D**). All of these results point to a strong inhibitory impact of DET on the growth of glioma cells.

### 3.7 DET induces glioma cells apoptosis

**Figure 6:**
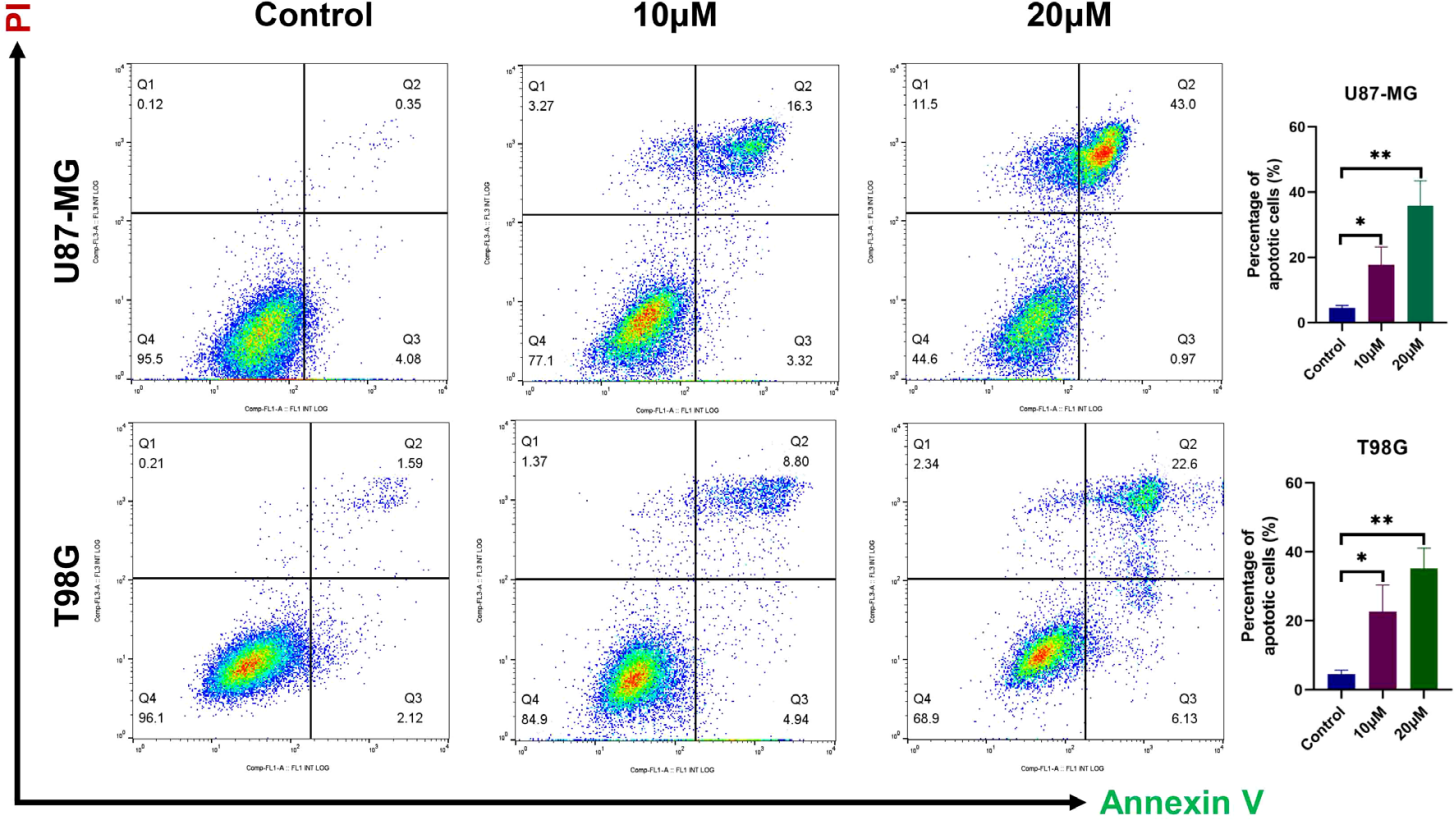
DET-induced glioma cell death was detected using flow cytometry. Before apoptosis was detected by a flow cytometer, U87-MG and T98G cells were treated with either vehicle (Control) or one of the indicated concentrations of DET (10 or 20 µM) for 24 hours. They were then harvested to create a single cell suspension and stained with 5 μL of Annexin V-FITC/PI double staining solution for 15 minutes at room temperature in the dark. *P<0.05, **P<0.01.

**Figure 7:**
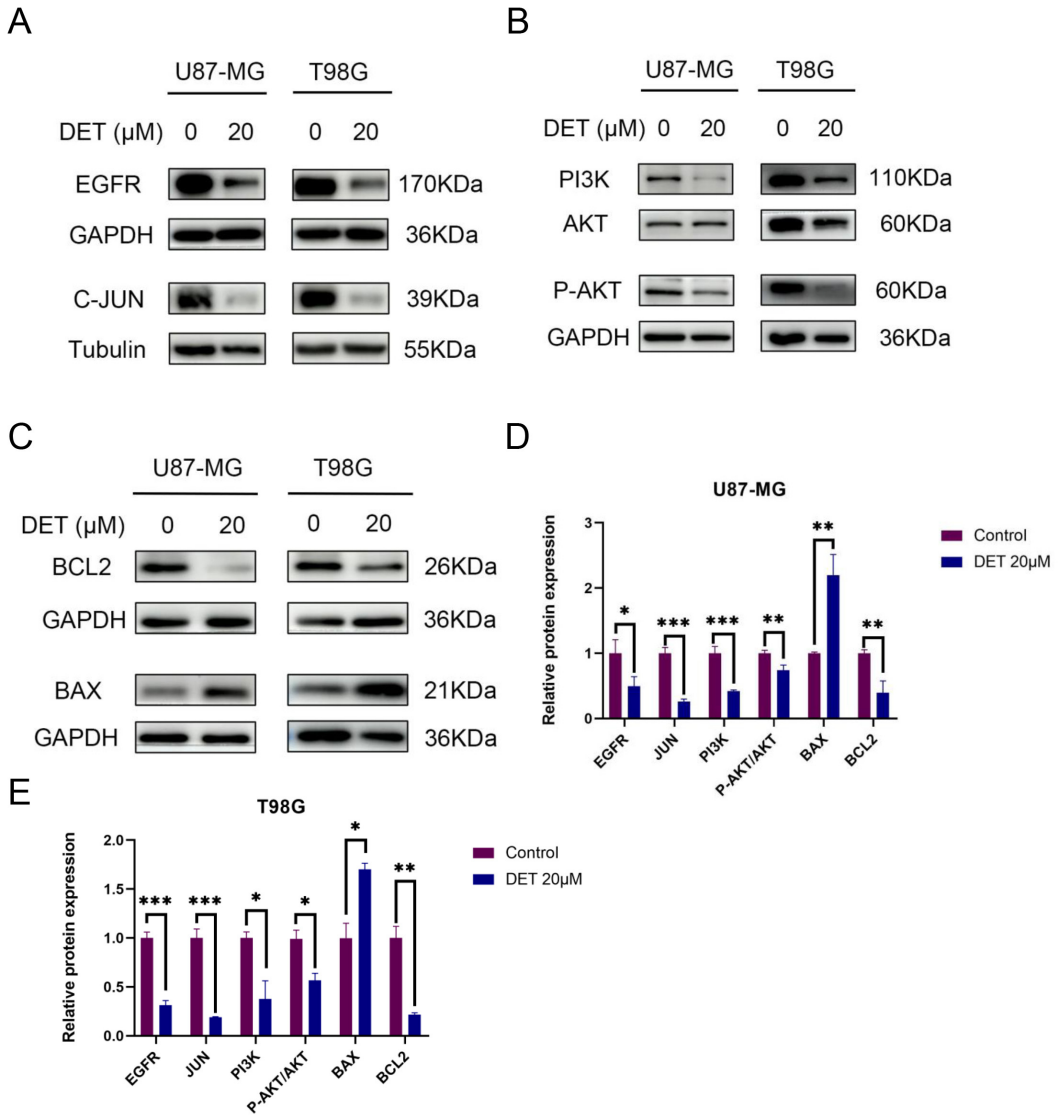
DET’s impact on U87-MG and T98G cells’ expression of key targets, the PI3K/AKT signaling pathway, and proteins linked to apoptosis. **(A)** Western blot experiment confirming that glioma cells treated with DET have lower expression levels of EGFR and C-JUN of DET's core targets. **(B)** According to experimental findings, DET reduced the glioma cells’ expression levels of the PI3K/AKT pathway. **(C)** The results of the Western blot indicate that DET may control glioma apoptosis by upregulating BAX expression levels and downregulating BCL2. **(D, E)** Individual protein expression levels in T98G and U87-MG cells following treatment with 20 μM DET, in comparison to control. *P<0.05, **P<0.01, ***P<0.001.

In order to investigate the pro-apoptotic effects of DET, different doses of DET were administered to U87-MG and T98G glioma cells, which were then stained using the Annexin V-FITC/PI double labeling procedure. The results indicated that both early and late apoptotic phases (Q2+Q3) in the DET-treated groups exhibited a significantly higher number of PI-positive cells, a marker of cell death (**Figure 6**). These findings suggest that DET induces apoptosis in a concentration-dependent manner in both the T98G and U87-MG glioma cell lines. Furthermore, DET modulates apoptosis in glioma cells by downregulating BCL2 expression while simultaneously upregulating BAX expression (**Figure 7C**). DET also affects glioma cell death by inhibiting the expression of EGFR/JUN (DET's core targets) and the core components of PI3K/AKT pathway (**Figures 7D, E**).

### 3.8 DET inhibits GBM cell invasion

**Figure 8:**
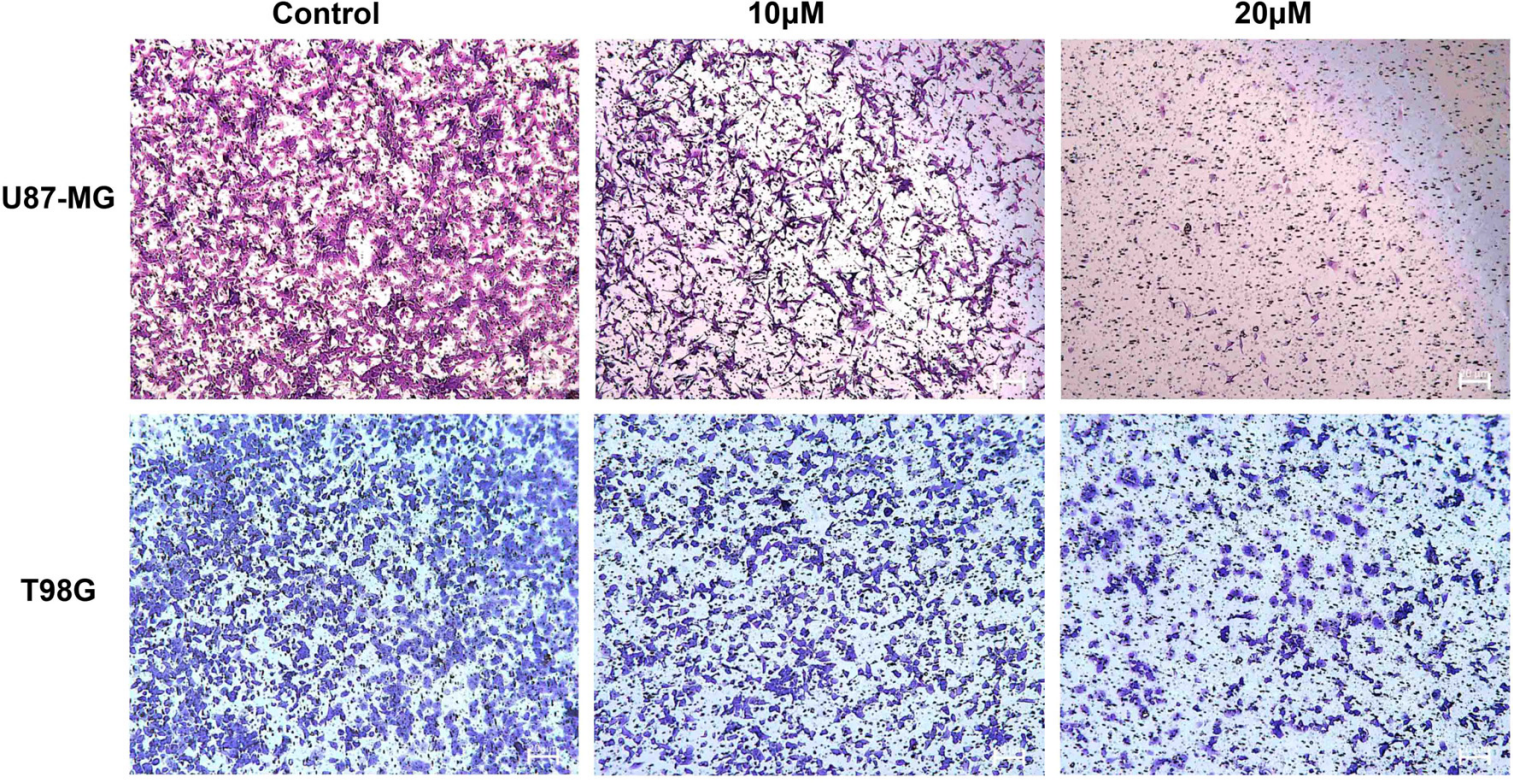
DET prevents the invasion of U87-MG and T98G cells. Transwell invasion experiment demonstrating dose-dependent inhibition of glioma cell invasion capacity by DET treatment.

A major factor in the development of gliomas and a major contributor to their high death rate is the invasive ability of tumor cells. The Transwell invasion experiment results showed that DET efficiently and dose-dependently inhibited the invasive behavior of T98G and U87-MG cell lines (**Figure 8**).

### 3.9 DET inhibits the PI3K/AKT signaling pathway in GBM cells

The expression levels of core proteins implicated in the core targets and signaling pathways were evaluated using Western blot analysis in conjunction with predictions of target involvement based on network pharmacology. EGFR, PI3K, and P-AKT expression levels were downregulated in both U87-MG and T98G GBM cell lines after a 24-hour DET therapy. Nevertheless, there was no discernible change in the overall levels of AKT. According to these results, DET may interfere with the growth and death of glioma cells via altering the PI3K/AKT signaling pathway (**Figures 7A, B**).

### 3.10 DET suppresses the tumor growth of the xenografted GBM cells

**Figure 9:**
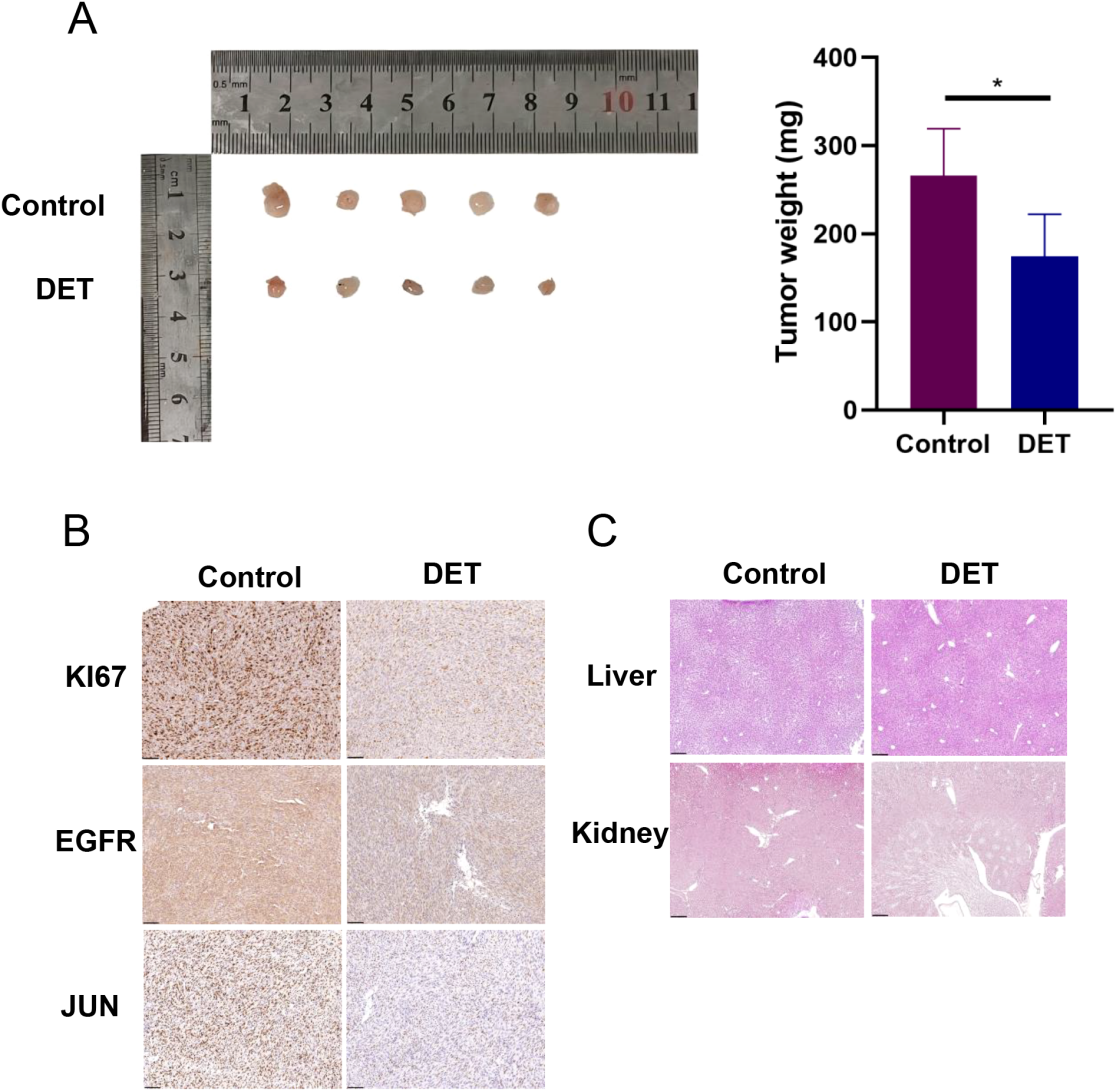
In nude mice, DET suppresses the growth of xenograft tumors and the expression of core targets. **(A)** In a xenograft tumor model of U87-MG cells, DET has an inhibitory impact on gliomas. **(B)** DET therapy suppressed the expression of EGFR, JUN, and KI67 in the transplanted tumors of naked mice, according to immunohistochemical staining results. **(C)** The findings of H&E staining of the kidney and liver of naked mice demonstrate that DET did not significantly harm these two organs.

The results from the animal experiments revealed that tumors in the control group were significantly larger than those in the DET-treated group (**Figure 9A**). Immunohistochemical analysis showed notable differences in the expression levels of KI67, EGFR, and JUN between the DET-treated and control groups (**Figure 9B**). However, histological examination using H&E staining showed no noticeable differences in liver and kidney morphology between the two groups, suggesting that DET did not cause damage to these vital organs involved in metabolism and excretion in the nude mice (**Figure 9C**).

## 4 Discussion

Despite extensive research, effective treatments that significantly prolong survival in glioma patients remain elusive. This is primarily due to the tumor’s rapid growth, high recurrence rate, and poorly defined boundaries between the tumor and normal tissue (27). Recently, the development of machine learning has enabled widespread application of bioinformatics in the diagnosis and treatment of diseases (28–31). To improve the prognosis and survival of GBM patients, this study explores novel therapeutic options. Through database searches and network pharmacology, 896 GBM-related targets and 264 DET-associated targets were identified. Subsequent construction of a protein-protein interaction (PPI) network, along with GO and KEGG enrichment analyses, highlighted EGFR and JUN as key prognostic targets for predicting five-year survival in glioma patients. *In vitro* experiments demonstrated that DET inhibited glioma cell invasion and proliferation in a dose-dependent manner. Western blot analysis confirmed that DET downregulated EGFR and JUN in glioma cells, while molecular docking further verified the binding of DET to these targets.

In GBM, EGFR is frequently overactive, leading to aberrant activation of the EGFR signaling pathway. This activation promotes tumor cell invasion and proliferation, contributing to the aggressive nature of the disease (32). EGFR promotes cell proliferation, anti-apoptosis, and chemotherapy resistance by activating the downstream PI3K/AKT and Ras/MAPK pathways (33). Cetuximab and other EGFR inhibitors, however, have a limited effectiveness in treating gliomas (34). This restriction emphasizes the need for improved therapies. Tumor cell migration is significantly influenced by JUN, which is up-regulated in GBM (35, 36).

KEGG analysis identified the PI3K/AKT pathway as a critical therapeutic target for DET. Our results further revealed that DET reduced the levels of phosphorylated AKT (P-AKT) and PI3K in both T98G and U87-MG cells. The PI3K/AKT pathway plays a pivotal role in regulating key processes such as cell growth, motility, angiogenesis, metabolism, and tumor cell survival (37, 38). Although only a few PI3K inhibitors are currently being tested in clinical trials for GBM, targeting this pathway remains critical for effective cancer treatment (39). Due to the recurrence and resistance of gliomas, single-target inhibitors of EGFR or PI3K/AKT often fail. Targeting both EGFR and PI3K/AKT simultaneously may offer a more effective therapeutic approach (40). Our findings show that DET inhibits glioma invasion and proliferation by downregulating EGFR, JUN, and PI3K/AKT. This suggests that DET may be a potential therapeutic for glioma by simultaneously targeting these critical proteins and signaling pathway involved in gliomagenesis.

Dysregulated apoptotic signaling allows cancer cells to evade cell death, facilitating unchecked growth and resistance to treatment (41, 42). Apoptosis is a critical mechanism in cancer therapy, with key therapeutic targets including anti-apoptotic proteins such as BCL2, which regulates mitochondrial membrane permeability to prevent apoptosis (43, 44). Our findings demonstrate that DET induces apoptosis in glioma cells by upregulating BAX and downregulating BCL2, consistent with previous studies in other malignancies (45, 46). This suggests that DET may possess anticancer properties (47). In a U87-MG xenograft model, DET inhibited tumor growth *in vivo* without affecting kidney or liver function. These *in vivo* results were further supported by immunohistochemical analysis, which revealed reduced expression of EGFR, JUN, and KI67 in the treated tumors.

These findings provide a foundation for future clinical investigations in neurosurgery, highlighting DET as a potential multi-targeted natural compound for glioma treatment. However, the lack of pharmacokinetic data and information on DET’s ability to cross the blood-brain barrier remains a limitation. Future studies will focus on evaluating the safety and efficacy of DET and its derivatives in intracranial xenograft models derived from glioma patients.

## 5 Conclusion

DET inhibits GBM cell invasion, proliferation, and apoptosis via modulating the PI3K/AKT signaling pathway and interacting with key targets, EGFR and JUN. This study provides a novel therapeutic strategy for GBM treatment and paves the way for future clinical drug development.

## Data Availability

The original contributions presented in the study are publicly available. This data can be found here: https://www.cgga.org.cn/download.jsp.
